# Illuminating Bilateral Breast Cancer: A Multicenter Experience and Clinical Observations

**DOI:** 10.3390/medicina61061029

**Published:** 2025-06-01

**Authors:** Berkan Karabuğa, Mustafa Büyükkör, Ekin Konca Karabuğa, Sedat Yıldız, Mirmehdi Mehtiyev, Havva Yeşil Çınkır, Sıla Soylu Koçoğlu, Hacer Demir, Ozan Yazıcı, Doğan Uncu, Ömür Berna Öksüzoğlu, Ülkü Yalçıntaş Arslan

**Affiliations:** 1 Department of Medical Oncology, Dr. Abdurrahman Yurtaslan Ankara Oncology Training and Research Hospital, Ankara 06200, Turkey; mbuyukkor@hotmail.com (M.B.); ulkuarslan63@gmail.com (Ü.Y.A.); 2 Department of Medical Oncology, Ankara Etlik City Hospital, Ankara 06170, Turkey; dr.ekinkk@gmail.com (E.K.K.); bernaoksuzoglu@yahoo.com (Ö.B.Ö.); 3 Department of Medical Oncology, Faculty of Medicine, Afyonkarahisar Health Sciences University, Afyonkarahisar 03030, Turkey; drsedatyildiz06@gmail.com (S.Y.); drhacerdemir@gmail.com (H.D.); 4 Department of Medical Oncology, Ankara Bilkent City Hospital, Ankara 06800, Turkey; mirmehdimehtiyev21@gmail.com (M.M.); doganuncu@yahoo.com (D.U.); 5 Department of Medical Oncology, Gaziantep University Faculty of Medicine, Gaziantep 27310, Turkey; doctoryesil82@yahoo.com; 6 Department of Medical Oncology, Gazi University Faculty of Medicine, Ankara 06560, Turkey; drsilasoylu@gmail.com (S.S.K.); drozanyazici@gmail.com (O.Y.)

**Keywords:** bilateral breast cancer, synchronous, metachronous

## Abstract

*Background and Objectives:* Although breast cancer is the most common type of cancer among women, bilateral breast cancer (BBC) remains exceedingly rare. BBC can present as either synchronous (SBBC) or metachronous (MBBC) disease. Data regarding the clinical characteristics of BBC are limited. In this study, we aimed to share our multicenter, retrospective experience on the clinicopathological and demographic features of SBBC and MBBC, their survival outcomes, and the factors influencing survival, in light of current knowledge. *Material and Method:* A total of 125 patients who were treated and followed between 2015 and 2024 across six different centers were included in the study. The patients were categorized into synchronous (SBBC) and metachronous (MBBC) groups. Their clinicopathological characteristics were analyzed, along with disease-free survival (DFS) and 5-year overall survival (OS) outcomes. *Results:* DFS was 5.7 years in the SBBC group and 5.6 years in the MBBC group (*p* = 0.95). The 5-year OS rate was 95.2% in the MBBC group and 80.7% in the SBBC group (*p* = 0.035). Hormone receptor negativity was identified as an independent risk factor for lower DFS in the overall patient cohort (HR: 0.55, 95% CI: 0.31–0.98, *p* = 0.04). *Conclusion:* Significant differences were found between the SBBC and MBBC groups in terms of hormone receptor status, presence of an invasive lobular carcinoma component, recurrence/metastasis status, and molecular subtype discordance between the two primary tumors. Although DFS did not significantly differ between the groups, the 5-year OS was significantly higher in the MBBC group. In multivariate regression analysis, hormone receptor negativity was identified as an independent risk factor for decreased DFS among all BBC patients. Our study is noteworthy for shedding light on the clinical features of BBC in the context of current knowledge and for its multicenter design.

## 1. Introduction

Although breast cancer is the most common type of cancer among women, bilateral primary breast cancer (BBC) is rare, with a reported incidence ranging from 1.4% to 11% [1]. BBC is classified as either synchronous (SBBC) or metachronous (MBBC), depending on whether the two distinct tumors are detected simultaneously or at different time points [2]. Patients diagnosed with breast cancer have a 2- to 6-fold increased risk of developing a new primary tumor in the contralateral breast compared to individuals without a history of breast cancer [3]. Pathogenic mutations such as BRCA1/2 and CHEK2 are believed to play a role in the pathogenesis of the disease. While the annual risk of developing a primary tumor in the contralateral breast is approximately 0.5% in individuals without such mutations, this risk increases to around 3% per year in BRCA mutation carriers [4,5]. The risk of BBC may also increase due to factors such as a younger age at the diagnosis of the first primary tumor, alcohol consumption, and tumor subtype [6]. In BBC, the risk of distant metastasis is higher compared to unilateral breast cancer, and this has been associated with reduced survival times relative to unilateral cases [7]. In patients with bilateral breast cancer (BBC), treatment planning is further complicated by factors such as the synchronous or metachronous development of tumors and the potential presence of differing histopathological and molecular subtypes in each breast [8]. Studies investigating the prognosis, treatment approaches, and survival outcomes of SBBC and MBBC bilateral breast cancer patients have demonstrated significant differences between these two subtypes. In a study conducted by Pan et al., the prognosis of patients with SBBC was reported to be worse than that of patients with unilateral breast cancer, whereas survival outcomes in MBBC patients were found to be comparable to those with unilateral disease [9].

Although numerous studies and clinical guidelines exist on breast cancer, there is a lack of studies providing clear data on the clinicopathological characteristics of BBC as well as evidence-based guidelines for its treatment and follow-up. Evidence-based treatment guidelines specific to BBC remain limited. Leading international guidelines such as those from the NCCN, CAP, and ESMO are primarily based on data from unilateral breast cancer and lack clear, specific recommendations for the management of BBC [10]. Therefore, further research focusing on BBC is essential to support optimal treatment decision making [11]. In our multicenter and retrospective study, we aimed to present the clinical, histopathological, and demographic characteristics of BBC, along with our survival outcomes, in light of current knowledge.

## 2. Materials and Methods

A total of 125 patients diagnosed with SBBC or MBBC, whose data were fully accessible, were included in our study from a pool of 134 patients treated and followed at six different centers between 2015 and 2024. Inclusion criteria were being diagnosed, treated, and followed up for BBC; age 18 years or older; and complete availability of clinical data. Exclusion criteria included incomplete data, age under 18 years, and the presence of an active secondary malignancy other than non-melanoma skin cancers. The clinical, histopathological, and demographic data of the patients were retrospectively analyzed. SBBC was defined as the diagnosis of a second primary tumor in the contralateral breast at the time of initial diagnosis, while MBBC was defined as the detection of a second primary tumor in the contralateral breast at any time following the diagnosis of the first primary tumor. SBBC and MBBC groups were compared in terms of these variables as well as survival outcomes, including overall survival (OS) and disease-free survival (DFS). Menopausal status was recorded for all patients, and tumor staging and grading were performed according to the 8th edition of the American Joint Committee on Cancer (AJCC) TNM staging system. Estrogen receptor (ER) and progesterone receptor (PR) statuses, assessed via immunohistochemistry at the time of diagnosis, were also retrospectively reviewed. ER expression greater than 1% was considered positive. At diagnosis, CERBB-2 (HER2) immunohistochemical staining was evaluated, with scores of 0 to 2 considered negative and a score of 3 considered HER2-positive. For patients with a CERBB-2 score of 2, HER2 status was verified using fluorescence in situ hybridization (FISH). Molecular subtype discordance was defined as any change in the immunohistochemical classification between the first and second primary tumors, such as a shift from HR+/HER2− to triple-negative, HR−/HER2+ to HR+/HER2−, or any other reclassification involving (ER/PR) or HER2 status. These data were retrospectively obtained from patient records. For both primary tumors, molecular subtype discordance based on hormone receptor and HER2 status, the presence of an invasive lobular carcinoma component at diagnosis, and the presence of local recurrence or distant metastasis were evaluated.

### Statistical Analysis

Statistical analyses were performed using the SPSS version 27.0 software package. Comparisons between groups were conducted using the chi-square test or Fisher’s exact test, as appropriate. Survival analyses were carried out using Kaplan–Meier curves. Variables found to be significant in univariate analysis were included in a multivariate Cox regression model. *p*-value of <0.05 was considered statistically significant. OS was defined as the time from the date of diagnosis to the date of death or last follow-up, while DFS was defined as the time from the diagnosis of the first primary tumor to the occurrence of local-regional recurrence or distant metastasis. For MBBC cases, survival durations were calculated based on the date of the first primary tumor diagnosis. For our study, multicenter ethical approval was obtained from the Non-Interventional Clinical Research Ethics Committee of the University of Health Sciences Dr. Abdurrahman Yurtaslan Ankara Oncology Training and Research Hospital, under the approval number: 2024-09/135.

## 3. Results

A total of 125 BBC patients (57 with SBBC and 68 with MBBC) who were treated and followed at six different centers between 2015 and 2024 were included in the study. All patients were female, with 45.6% in the SBBC group and 54.4% in the MBBC group. The clinicopathological and demographic characteristics of the patients are presented in Table 1. Of the patients, 53.6% were under the age of 50, while 46.4% were aged 50 or older. The median follow-up period was 6.02 years (range: 0.78–29.6 years). ER positivity was observed in 76.8% of all BBC patients; when analyzed by subgroup, ER positivity was 91.2% in the SBBC group and 64.7% in the MBBC group (*p* < 0.001). PR positivity was 87.7% in the SBBC group and 66.2% in the MBBC group (*p* = 0.005). The presence of local recurrence or metastasis was significantly higher in the MBBC group (82.4%) compared to the SBBC group (36.8%) (*p* < 0.001). At diagnosis, the presence of an invasive lobular carcinoma component was found in 35.0% of SBBC cases and 10.3% of MBBC cases (*p* = 0.001). Molecular subtype discordance between the two primary tumors was identified in 51.5% of MBBC cases and 26.3% of SBBC cases (*p* = 0.006).

Histological and immunohistochemical subtypes of the first and second primary tumors in the SBBC and MBBC groups are presented in Table 2. Accordingly, invasive ductal carcinoma was the most common histological subtype in both the first and second primary tumors across both groups (SBBC: 34 [59.6%] and 33 [57.9%]; MBBC: 51 [75.0%] and 41 [60.3%], respectively). In terms of immunohistochemical subtypes, HR(+)/HER2(+) tumors were more frequently observed than other subtypes in both the first and second primary tumors of each group (SBBC: 37 [64.9%] and 41 [71.9%]; MBBC: 37 [54.4%] and 34 [50.0%], respectively).

**Figure 1:**
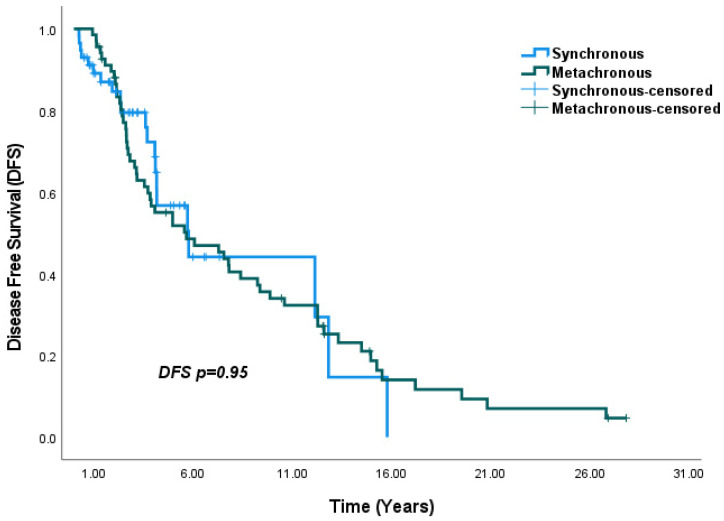
DFS in Synchronous and Metachronous Breast Cancer: No significant difference in DFS was observed between the SBBC and MBBC groups.

**Figure 2:**
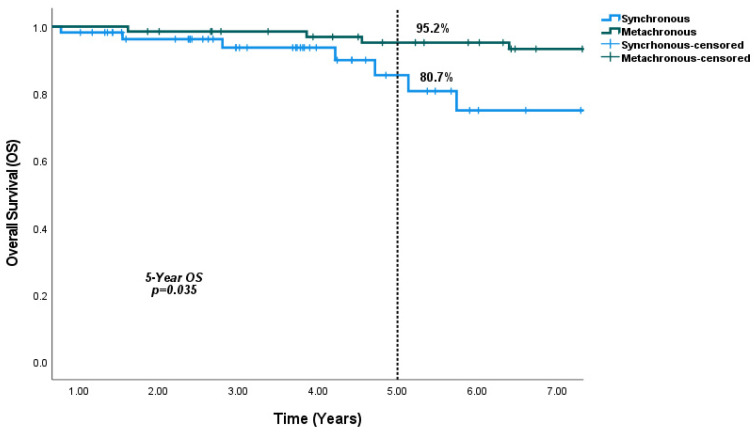
5-Year OS Rates in Synchronous and Metachronous Breast Cancer: 5-year OS was found to be significantly higher in the MBBC group compared to the SBBC group.

In terms of survival outcomes, DFS was 5.7 years in SBBC group and 5.6 years in the MBBC group, with no significant difference observed (*p* = 0.95) (Figure 1). However, the 5-year OS rate was significantly higher in the MBBC group (95.2%) compared to the SBBC group (80.7%) (*p* = 0.035) (Figure 2).

Multivariate Cox regression analysis revealed that hormone receptor negativity was an independent risk factor for reduced DFS in the overall BBC population (HR: 0.57, 95% CI: 0.33–0.98, *p* = 0.04) (Table 3).

**Figure 3:**
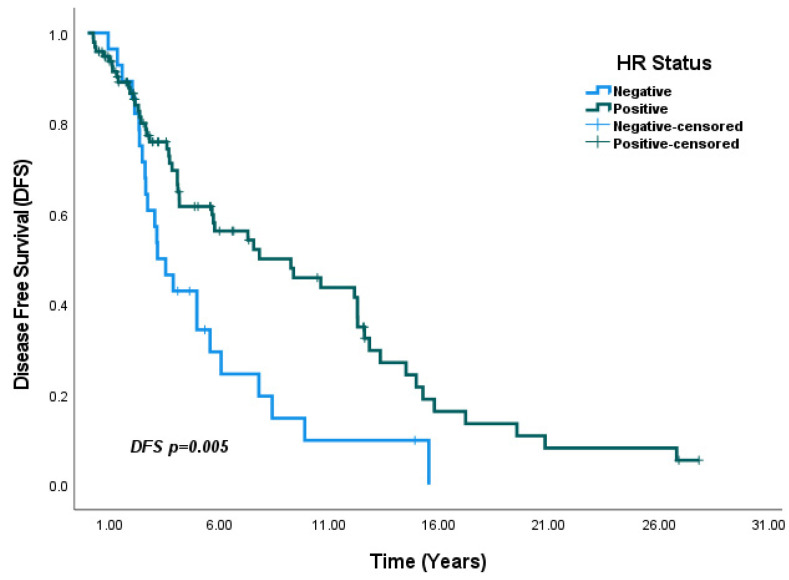
DFS according to HR status in all BBC patients: DFS was significantly higher in the HR-positive group compared to the HR-negative group (*p* = 0.005).

**Figure 4:**
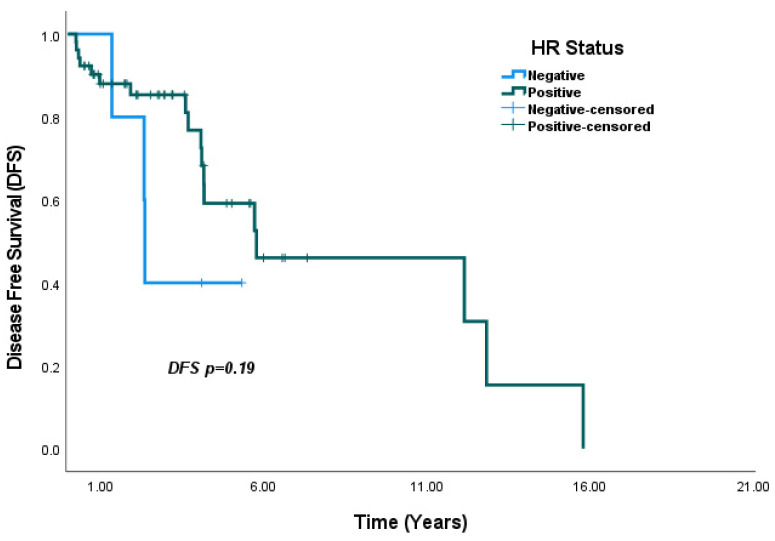
Analysis of DFS by HR status within the SBBC group: Although not statistically significant, DFS was numerically higher in HR-positive patients compared to HR-negative patients (*p* = 0.19).

**Figure 5:**
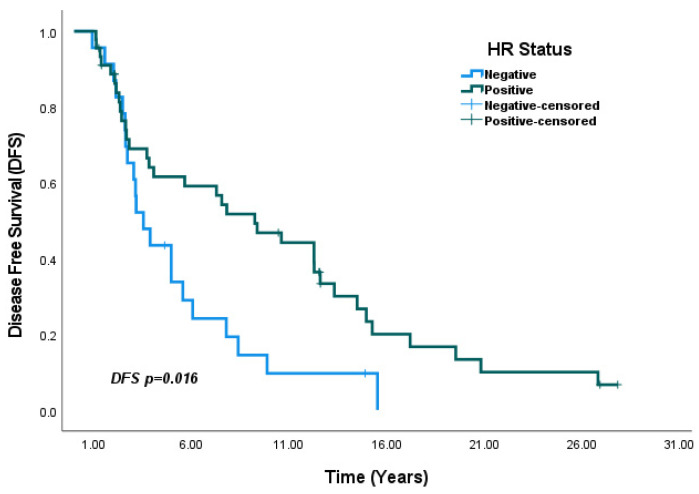
Analysis of DFS by HR status within the MBBC group: DFS was significantly higher in HR-positive patients compared to HR-negative patients (*p* = 0.016).

In the overall population, DFS analysis based on HR status showed that median DFS was significantly higher in HR-positive patients compared to HR-negative patients (7.8 years vs. 3.1 years, *p* = 0.005) (Figure 3). In subgroup analyses by HR status, median DFS was numerically higher in HR-positive patients than in HR-negative patients within the SBBC group, although the difference was not statistically significant (5.7 years vs. 2.3 years, *p* = 0.19) (Figure 4). In contrast, in the MBBC group, median DFS was significantly higher in HR-positive patients compared to HR-negative patients (9.2 years vs. 3.5 years, *p* = 0.016) (Figure 5).

## 4. Discussion

Although breast cancer is the most commonly diagnosed cancer in women, BBC remains a rare clinical entity. In the literature, various definitions have been used to differentiate SBBC from MBBC. While some studies define SBBC as the simultaneous detection of both primary tumors, others define it based on the diagnosis of the second primary tumor within one month or six months of the first. In our study, SBBC was defined as the concurrent detection of both primary tumors, while MBBC referred to cases where the tumors were diagnosed at different time points [1,12,13]. The majority of patients in our study belonged to the MBBC group. This is consistent with previous studies that have reported a higher incidence of MBBC compared to SBBC [14,15,16]. However, some studies have reported similar frequencies or even a higher incidence of SBBC [17,18]. These discrepancies may stem from differing definitions across studies, improved detection of SBBC through high-sensitivity imaging modalities such as MRI, and the increased incidence of MBBC due to a higher risk of contralateral tumors in unilateral breast cancer patients over prolonged follow-up periods [19]. These findings support the need for future studies to adopt standardized international criteria for defining SBBC and MBBC in order to improve patient management and reduce inconsistencies across study outcomes. They also highlight the importance of encouraging the use of more sensitive diagnostic modalities, such as MRI, in the evaluation of patients with suspected BBC. Furthermore, systematic and prolonged surveillance of the contralateral breast should be considered in high-risk breast cancer patients (e.g., those with TNBC or early-onset disease).

### 4.1. Clinicopathological and Histological Characteristics

A study investigating the clinicopathological characteristics of SBBC and unilateral breast cancer reported higher ER positivity in BBC patients. Similarly, our study found high ER positivity across all BBC cases [20]. In line with our findings, Shi et al. reported ER-positivity rates above 70% in BBC patients [21]. In our study, ER and PR positivity were significantly higher in the SBBC group compared to the MBBC group, while HER2 status did not differ significantly. This aligns with findings by Hong et al., who also reported significantly higher ER and PR positivity in SBBC cases (97.2% and 49.5%, respectively) and noted that even when the first tumor was HR-negative, the second often showed positivity [18]. Verkooijen et al. reported a trend toward HR negativity in MBBC cases [19]. These results emphasize the importance of hormone therapy in preventing contralateral primary tumors, particularly in unilateral luminal-type breast cancer.

In our cohort, the presence of an invasive lobular carcinoma (ILC) component was significantly higher in SBBC compared to MBBC. ILC is less common than invasive ductal carcinoma (IDC) and typically presents with lower histological grade and a more indolent clinical course [22]. Li et al. suggested ILC may be predictive of BBC development [2]. A Danish study comparing SBBC with unilateral breast cancer also found a significantly higher rate of ILC in SBBC cases [20]. Verkooijen et al. reported similar findings, suggesting ILC may contribute to the development of contralateral tumors [19]. These findings underscore the need for more detailed assessment and close follow-up of patients diagnosed with unilateral ILC, ideally using advanced diagnostic tools.

### 4.2. Hormone Receptor Status and IHC Subtype

HR status is crucial in defining the tumor’s immunohistochemical subtype. Luminal tumors generally have a better prognosis compared to non-luminal ones [23]. The risk of developing a second primary tumor in the contralateral breast is higher in HR-negative cases, while true metastasis to the contralateral breast is rare [19]. In our study, HR negativity emerged as an independent risk factor for reduced DFS. The DFS analyses conducted in all BBC patients as well as in the SBBC and MBBC subgroups consistently support this finding, demonstrating that DFS is higher in HR-positive patients compared to HR-negative ones. Similarly, in a study of 123 BBC patients by Hong et al., HR negativity was also linked to poorer DFS [18]. Kheirelseid et al. likewise noted that HR negativity increased the risk of contralateral tumor development [24].

Our study further revealed significantly greater immunohistochemical subtype discordance between the tumors in MBBC cases compared to SBBC. The higher frequency of HR negativity and IHC subtype discordance in MBBC supports the theory that these represent independent primary tumors rather than metastases from the first tumor. This biological behavior may also contribute to increased recurrence and metastasis risk, correlating with poorer DFS. Based on this, any contralateral lesion detected in a patient with unilateral breast cancer should not be presumed metastatic but instead be pathologically confirmed via biopsy to rule out a second primary tumor.

### 4.3. Survival Outcomes

Our findings showed that local recurrence and metastasis were significantly more frequent in MBBC patients than in SBBC patients. Baykara et al. similarly found a 52.2% distant metastasis rate in MBBC, with both local and distant recurrences being significantly more common than in SBBC cases (*p* < 0.0001) [1]. Vuoto et al. also observed higher rates of distant metastasis and local recurrence in MBBC, although these differences were not statistically significant [14].

In our study, the 5-year OS was significantly higher in MBBC compared to SBBC, while DFS did not differ significantly. In contrast, Baykara et al. reported a 5-year OS of 90% in both groups. Beckmann et al. found similar results to ours, with OS of 87.3% in MBBC and 79.3% in SBBC [1,25]. Verkooijen et al. reported 5-year disease-specific survival of 77% for SBBC and 80% for MBBC, and 10-year survival rates of 51% and 66%, respectively [19]. Kollias et al. reported 16-year OS rates of 42.4% for SBBC and 60.1% for MBBC [26]. Takahashi et al. found no OS difference between the groups, with 10-year DFS rates of 65% for MBBC and 64.3% for SBBC [27]. Hong et al. reported 5-year OS rates of 91% for SBBC and 95% for MBBC, with no significant DFS difference [18].

These inconsistencies in the literature may be attributed to factors such as inclusion of in situ cases, varying cohort sizes, and differences in follow-up duration. Some studies lack the statistical power to detect OS differences due to small sample sizes. Additionally, confounding variables such as age, disease stage, and evolving treatment protocols may not have been accounted for. The inclusion of patients across a wide age range and all disease stages may influence the interpretation of the results, as both age and disease stage are well-known prognostic factors in breast cancer. For instance, younger patients (particularly those under 40) tend to present with more aggressive tumor biology and have a higher risk of local recurrence, which may negatively impact survival outcomes [28]. In contrast, older patients (>70 years) often exhibit less aggressive tumor features but may experience worse overall survival due to treatment-related toxicity and comorbid conditions; similarly, disease stage significantly affects survival and treatment response. Patients diagnosed at early stages generally have more favorable outcomes, whereas those with advanced-stage disease show poorer survival rates [29].

In our study, the significantly higher OS in MBBC despite comparable DFS may reflect the use of modern therapies including CDK4/6 inhibitors and novel anti-HER2 agents, which prolong survival. For example, a real-world study reported a 3-year OS rate of 73.0% for patients receiving endocrine therapy combined with a CDK4/6 inhibitor compared to 49.1% for those receiving endocrine therapy alone (log-rank *p* < 0.0001) [30]. Similarly, HER2-targeted therapies have significantly improved survival outcomes in HER2-positive breast cancer. The combination of docetaxel, pertuzumab, and trastuzumab has been associated with a median survival exceeding 4.5 years, a notable improvement from the historical median of 1.5 years [31]. Additionally, newer antibody–drug conjugates have further enhanced treatment outcomes. Trastuzumab emtansine (T-DM1) has shown improved OS in HER2-positive early breast cancer patients with residual invasive disease after neoadjuvant therapy; in the KATHERINE trial, 7-year OS was 89.1% with T-DM1 versus 84.4% with trastuzumab [32]. Trastuzumab deruxtecan (T-DXd) has demonstrated superior efficacy over T-DM1 in HER2-positive metastatic breast cancer, with median OS of 52.6 months in the T-DXd arm versus 42.7 months in the T-DM1 arm, and the 36-month PFS rate was 45.7% versus 12.4%, respectively, as shown in the DESTINY-Breast03 trial [33]. Furthermore, sacituzumab govitecan significantly improved survival in patients with metastatic triple-negative breast cancer; in the ASCENT trial, median OS was 12.1 months with sacituzumab govitecan versus 6.7 months with chemotherapy [34]. Differences in treatment over time, patient classification criteria, and inclusion criteria may also play roles. For example, some patients we classified as MBBC may have been considered SBBC in earlier studies. Access to modern local therapies and variation in recurrence sites might also contribute. Differences in tumor burden among MBBC patients may have influenced the OS estimates in this group. The observed difference between OS and DFS in the MBBC group may, in part, be attributed to methodological factors such as lead-time and guarantee-time biases. In MBBC patients, survival is typically calculated from the time of diagnosis of the first tumor, which may artificially prolong OS due to earlier detection and initiation of follow-up, an effect known as lead-time bias [35]. Moreover, MBBC patients, by definition, must survive long enough to develop a second primary tumor, introducing a guarantee-time bias that may result in overestimation of OS compared to SBBC cases [36]. These biases should be considered when interpreting survival differences between the two groups.

### 4.4. Implications and Recommendations

The higher rates of local recurrence and distant metastasis observed in the MBBC group suggest that follow-up for these patients should be conducted more frequently and systematically. Given these elevated recurrence and metastasis rates, further investigation into genetic mutations, microsatellite instability, and tumor heterogeneity is warranted to provide insights that may guide the management of this subgroup. The differences between DFS and OS, particularly in MBBC cases, highlight the potential impact of novel therapeutic agents on overall survival. In addition to improving survival outcomes, enhancing the quality of life in this patient population remains a critical aspect of clinical care. To better understand the differences in the management of SBBC and MBBC and facilitate their implementation in clinical practice, future studies should incorporate subgroup-based prospective evaluations. Moreover, real-world data analyses are needed to elucidate the discrepancies between DFS and OS.

### 4.5. Study Strengths and Novel Contributions

In light of these findings, it is also important to highlight the unique aspects of our study design and population. This study offers a unique contribution to the literature by evaluating a relatively large cohort of BBC patients, with a direct comparison between SBBC and MBBC subtypes using uniform diagnostic criteria. Unlike many previous studies that rely on heterogeneous definitions and limited subgroup analyses, our study applies a standardized classification and includes patients across all disease stages. Furthermore, by incorporating both histopathological and molecular subtype discordance along with long-term survival data, our methodology allows for a more comprehensive understanding of clinical and prognostic differences between SBBC and MBBC. These features distinguish our study from prior work and provide valuable insights for both current clinical practice and future research planning.

### 4.6. Limitations

Our study has several limitations. First, the lack of consensus on SBBC and MBBC definitions affects classification and comparability. Second, all BBC patients were included regardless of disease stage, leading to heterogeneous treatment approaches, especially in metastatic cases. Third, BRCA status was unknown in most cases, limiting analysis of genetic predisposition. Lastly, the high proportion of censored data may affect the robustness of survival analyses. To address these limitations in future research, we recommend the adoption of standardized definitions for SBBC and MBBC to improve comparability across studies. Stratified analyses based on disease stage should be considered to minimize treatment heterogeneity. Incorporating genetic testing, including BRCA mutation status, would allow for more accurate assessment of hereditary risk factors. Finally, efforts to reduce censored data through longer follow-up periods and prospective study designs would enhance the reliability of survival analyses.

## 5. Conclusions

BBC is a rare condition, and to date, there is a lack of clear data regarding its clinicopathological characteristics, treatment, and follow-up algorithms. In our study, HR positivity and the presence of an invasive lobular carcinoma component were significantly higher in the SBBC group compared to the MBBC group. Conversely, the MBBC group demonstrated significantly higher rates of local recurrence and metastasis, molecular subtype discordance between the two breasts, and 5-year OS compared to SBBC group. While OS data are not yet fully mature, no significant difference in DFS was observed between the two groups. HR negativity was identified as an independent risk factor for reduced DFS across the entire patient cohort. Our study is noteworthy for being a multicenter investigation that provides insight into the clinical, histopathological, and demographic features of BBC in the context of current knowledge and treatment modalities.

## Figures and Tables

**Table 1:** Clinicopathological and Demographic Characteristics of the Patients. ER/PR status: ER/PR positivity, molecular subtype discordance, presence of an invasive lobular carcinoma component, and occurrence of recurrence/metastasis were found to be significantly higher in the MBBC group compared to the SBBC group.

	SBBC n: 57 (%)	MBBC n: 68 (%)	*p*-Value
Age			
<50	29 (50.9)	38 (55.9)	0.576 ^1^
≥50	28 (49.1)	30 (44.1)	
Menapousal Status			
Premenapousal	25 (43.9)	33 (48.5)	0.602 ^1^
Postmenapousal	32 (56.1)	35 (51.5)	
T Stage			
T1–2	43 (75.4)	51 (75)	0.955 ^1^
T3–4	14 (24.6)	17 (25)	
Nodal Status at Diagnosis			
Negative	17 (29.8)	29 (42.6)	0.139 ^1^
Positive	40 (70.2)	39 (57.4)	
ER Status at Diagnosis			
ER (-)	5 (8.8)	24 (35.3)	<0.001 *^1^
ER (+)	52 (91.2)	44 (64.7)	
PR Status at Diagnosis			
PR (-)	7 (12.3)	23 (33.8)	0.005 *^1^
PR (+)	50 (87.7)	45 (66.2)	
HER2 Status at Diagnosis			
HER2 (-)	39 (68.4)	44 (64.7)	0.66 1 ^1^
HER2 (+)	18 (31.6)	24 (35.3)	
Immunohistochemical (IHC) Subtype Discordance (Between the Two Breast Cancers)			
None	40 (70.2)	29 (42.6)	0.006 *^2^
Yes	15 (26.3)	35 (51.5)	
Unknown	2 (3.5)	4 (5.9)	
Presence of Invasive Lobular Carcinoma Component at Diagnosis			
Negative	36 (63.2)	54 (79.4)	0.001 *^2^
Positive	20 (35.0)	7 (10.3)	
Unknown	1 (1.8)	7 (10.3)	
Recurrens/Metastasis Status			
None	36 (63.2)	12 (17.6)	<0.001 *^1^
Yes	21 (36.8)	56 (82.4)	
Grade at Diagnosis			
Grade 1	9 (16.1)	3 (4.4)	0.087 ^1^
Grade 2	29 (51.8)	38 (55.9)	
Grade 3	18 (32.1)	27 (39.7)	

* *p* < 0.05; ^1^ chi-square test; ^2^ Fischer exact test.

**Table 2:** Histological and immunohistochemical subtype distribution of the first and second primary tumors in SBBC and MBBC patients: Invasive ductal carcinoma and HR(+)/HER2(+) subtype were the most common findings in both groups.

	SBBC n: 57 (%)	MBBC n: 68 (%)
First Primer Tumor Histology		
Invasive Ductal Carcionma	34 (59.6)	51 (75.0)
Invasive Lobular Carcinoma	14 (24.6)	6 (8.8)
Mix Type	2 (3.5)	2 (2.9)
Other	7 (12.3)	9 (13.2)
Second Primer Tumor Histology		
Invasive Ductal Carcinoma	33 (57.9)	41 (60.3)
Invasive Lobular Carcinoma	8 (14.0)	6 (8.8)
Mix Type	2 (3.5)	0 (0.0)
Other	14 (24.6)	21 (30.9)
First Primer Tumor Subtype (IHC)		
HR (+)/HER2 (+)	13 (22.8)	12 (17.6)
HR (+)/HER2 (-)	37 (64.9)	37 (54.4)
HR (-)/HER2 (+)	5 (8.8)	8 (11.8)
TNBC	2 (3.5)	11 (16.2)
Unknown	0 (0.0)	0 (0.0)
Second Primer Tumor Subtype (IHC)		
HR (+)/HER2 (+)	9 (15.8)	7 (10.3)
HR (+)/HER2 (-)	41 (71.9)	34 (50.0)
HR (-)/HER2 (+)	4 (7.0)	9 (13.2)
TNBC	1 (1.8)	14 (20.6)
Unknown	2 (3.5)	4 (5.9)

**Table 3:** Multivariate Cox Regression Analysis for DFS: HR negativity was identified as an independent risk factor for DFS.

	Univariate Cox Regression		Multivariate Cox Regression	
	HR (CI 95%)	*p*-Value	HR (CI 95%)	*p*-Value
<50 age vs. ≥50 age	1.23 (0.78–1.93)	0.363		
Premenopausal vs. Postmenopausal	0.69 (0.44–1.09)	0.11		
SBBC vs. MBBC	1.01 (0.59–1.71)	0.95		
HR negative vs. HR positive	0.49 (0.30–0.82)	<0.01	0.57 (0.33–0.98)	0.04 *
CerbB2 negative vs. CerbB2 positive	1.73 (1.07–2.79)	0.02	1.43 (0.85–2.40)	0.17

* *p* < 0.05.

## Data Availability

Dataset available on request from the authors.

## Abbreviations

BBC	Bilateral Breast Cancer
SBBC	Synchronous Breast Cancer
MBBC	Metachronous Breast Cancer
DFS	Disease-Free Survival
OS	Overall Survival
ER	Estrogen Receptor
PR	Progesterone Receptor
HR	Hormone Receptor
CHEK2	Checkpoint Kinase 2
FISH	Fluorescence In Situ Hybridization
